# Unusual manifestation of fungal malignant external otitis

**DOI:** 10.11604/pamj.2020.36.337.20814

**Published:** 2020-08-25

**Authors:** Mafalda da Silva Ferreira, Clara Silva

**Affiliations:** Otolaryngology (ENT) Department, Coimbra University Hospital, Coimbra, Portugal

**Keywords:** External otitis, malignant external otitis, fungal external otitis

## Image in medicine

Malignant external otitis (MEO) is a fatal disease of the external auditory canal and temporal bone. The infection begins as an external otitis that later spreads and turns into an osteomyelitis of the temporal bone. It has an extremely aggressive behavior and poor prognosis. It mainly affects immunocompromised men over 60 years old. Diabetes is the most common risk factor and *Pseudomonas Aeruginosa* is the organism responsible for 90% of all cases. These pictures represent a case of an extremely invasive fungal MEO in a 59 years old man. It was a patient with a history of MEO on the right ear, with a poorly controlled type II diabetes and a chronic renal insufficiency. He presented with one month complaint of severe left ear pain and discharge. In otoscopy (A) we observed otorrhea and granulation tissue at the osseocartilaginous junction. Swab culture from the ear canal showed the presence of *Aspergillus Flavus*, an uncommon organism. Technetium and gallium scans reveled an intense uptake within the left temporal bone. During the treatment with intravenous voriconazole and vancomycin the patient developed paresis of VI nerve (B). Head MRI showed significant inflammatory soft tissue in mastoids, with an extensive inflammatory process involving the skull base (C). Cranial nerves can be damage when the infection spreads along skull base. The patient remains under antifungal treatment and with a special concern about pain relieving.

**Figure 1:**
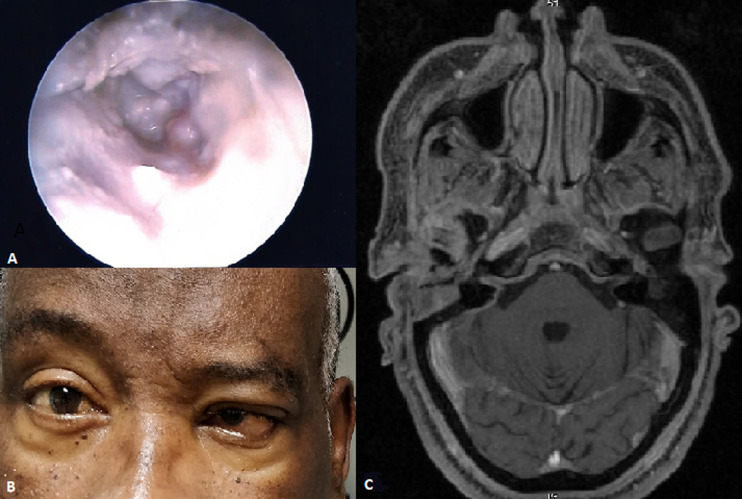
(A,B,C) fungal malignant external otitis

